# HB-EGF Improves the Hair Regenerative Potential of Adipose-Derived Stem Cells via ROS Generation and Hck Phosphorylation

**DOI:** 10.3390/ijms21010122

**Published:** 2019-12-23

**Authors:** Nahyun Choi, Won-Serk Kim, Sang Ho Oh, Jong-Hyuk Sung

**Affiliations:** 1 College of Pharmacy, Yonsei Institute of Pharmaceutical Sciences, Yonsei University, Incheon 21983, Korea; nh147837@gmail.com; 2 STEMORE Co. Ltd., Incheon 21983, Korea; 3 Department of Dermatology, Kangbuk Samsung Hospital, Sungkyunkwan University School of Medicine, Seoul 03181, Korea; susini@naver.com; 4 Department of Dermatology, Severance Hospital and Cutaneous Biology Research Institute, Yonsei University College of Medicine, Seoul 03722, Korea; ODDUNG93@yuhs.ac

**Keywords:** adipose-derived stem cell, HB-EGF, hair growth, THPO, dermal papilla cell, reactive oxygen species, Hck phosphorylation

## Abstract

Although adipose-derived stem cells (ASCs) have hair regenerative potential, their hair inductive capabilities are limited. The mitogenic and hair inductive effects of heparin binding-epidermal growth factor-like growth factor (HB-EGF) on ASCs were investigated in this study and the underlying mechanism of stimulation was examined. Cell growth, migration, and self-renewal assays, as well as quantitative polymerase chain reactions and immunostaining, were carried out. Telogen-to-anagen transition and organ culture using vibrissa follicles were also conducted. HB-EGF significantly increased ASC motility, including cell proliferation, migration, and self-renewal activity. The preconditioning of ASCs with HB-EGF induced telogen-to-anagen transition more rapidly in vivo, and injected PKH26-ASCs survived for longer periods of time. Conditioned medium obtained from HB-EGF-treated ASCs promoted hair growth in vivo, upregulating growth factors. In particular, thrombopoietin (THPO) also induced hair growth in vivo, stimulating dermal papilla cells (DPCs). Reactive oxygen species (ROS) appeared to play a key role in ASC stimulation as the inhibition of ROS generation and NOX4 knockout attenuated ASC stimulation and THPO upregulation by HB-EGF. In addition, the Hck phosphorylation pathway mediated the stimulation of ASCs by HB-EGF. In summary, HB-EGF increased the motility and paracrine effects of ASCs releasing THPO growth factor and THPO promoted hair growth-stimulating DPCs. ROS generation and Hck phosphorylation are key factors in HB-EGF-induced ASC stimulation. Therefore, combination therapy involving HB-EGF and ASCs may provide a novel solution for hair-loss treatment.

## 1. Introduction

Our previous studies demonstrated that adipose-derived stem cells (ASCs) have diverse skin and hair regenerative potential [1,2]. Although the injection of ASCs and a conditioned medium of ASCs (ASC-CM) promoted the telogen-to-anagen transition in an animal model, their regenerative potential remains unsatisfactory [3,4]. For example, anagen induction by ASCs or ASC-CM is not as effective as induction by minoxidil. Consequently, several studies have been conducted to identify ASC stimulators. ASCs can be stimulated with LL-37 to improve their paracrine effects and hair regenerative potential [5]. Additionally, hypoxia and low-dose reactive oxygen species (ROS) promote ASC proliferation and the secretion of growth factors by ASCs, and ASC-CM obtained under hypoxia was reported to enhance the telogen-to-anagen transition in vivo [6,7]. Platelet-derived growth factor-D (PDGF-D) generates mitochondrial ROS to increase mitochondrial fission and ASC proliferation [8]. Though PDGF-D-preconditioned ASCs show enhanced hair regenerative potential in vivo, there remains a need to further improve the hair inductivity of ASCs.

Our previous study aimed to differentiate ASCs into dermal papilla cells (DPCs) via the transfection of three trichogenic genes, namely PDGF-A, Sox2, and β-catenin [9]. mRNAseq was performed to compare the global gene expression profiles of the ASCs, three gene-transfected ASCs (tfASCs), and DPCs. Amphiregulin and epiregulin are expressed at high levels in tfASCs and DPCs [9]. As these molecules are epidermal growth factor (EGF) mimetics, we hypothesized that recombinant proteins of amphiregulin, epiregulin, or other EGF mimetics, such as heparin binding-EGF-like growth factor (HB-EGF), could differentiate or stimulate ASCs to enhance hair inductivity. Among these EGF mimetics, HB-EGF had the best effect on ASC proliferation and migration. Therefore, the hair growth function of HB-EGF was focused on in this study. It was first checked whether HB-EGF treatment can differentiate ASCs into DPCs. When HB-EGFs are treated for long periods of time, ASCs change their motility, but do not differentiate into DPCs, by confirming cell morphology and the expression of DPC marker genes. HB-EGF treatment did not affect ASC morphology and the expression level of the DPC marker genes (data not shown). Therefore, we focused on whether HB-EGF can stimulate ASC motility and their hair growth effect.

EGF and receptor tyrosine kinases of the EGF family (ErbBs) are essential in regulating cell proliferation, survival, differentiation, and migration. EGF assists in epidermal layer regeneration and is commonly used as a raw material in cosmetics, as well as to treat diabetic foot ulcers [10]. EGF is involved in cancer progression and both inhibitors and antibodies targeting ErbBs have been developed to treat various cancer types [11]. It was reported that EGFR signaling is indispensable for the initiation of hair growth, and the continuous expression of EGF prevents entry into the catagen phase [12]. In addition, EGF promotes the proliferation and migration of follicular outer root sheath cells via Wnt/β-catenin signaling [13]. However, whether EGF mimetics, such as HB-EGF, exert effects on hair growth or hair cycling has not been investigated.

The ligand specificity, redundancy, processing, variable tissue expression patterns, and signaling diversity of the EGF pathway have been well-reported. The mitogen-activated protein kinase (MAPK), STAT, and PI3K/Akt pathways are activated following EGF receptor (EGFR) phosphorylation. Between the phosphorylation of ErbB and these signaling molecules, SRC families play key roles in mediating the signaling pathways. EGF binding to its receptor causes rapid phosphorylation of the clathrin heavy chain at tyrosine 1477, which lies in a domain controlling clathrin assembly [14]. In cells lacking SRC kinase, or cells treated with a specific SRC family kinase inhibitor, EGF stimulation of clathrin phosphorylation and redistribution does not occur, and EGF endocytosis is delayed [14]. These observations demonstrate a role for SRC kinase in the modification and recruitment of clathrin during ligand-induced EGFR endocytosis. In our study, to elucidate novel receptor tyrosine kinase (RTK) binding with HB-EGF, we carried out an RTK assay. We found that Hck, which is one of the SRC family kinases, was phosphorylated after HB-EGF treatment, in addition to EGFR1. However, there is little to no evidence that Hck is associated with the ErbB and mitogenic signal pathways.

Although the basic fibroblast growth factor (bFGF) and PDGF families play key roles in the self-renewal, proliferation, and paracrine effects of ASCs, there have been several reports on the effects of EGF families on the proliferation and differentiation of ASCs during culturing. For example, EGF treatment promoted the proliferation and maintained the multipotency of continuously cultured ASCs via activating the STAT-signaling pathway in vitro [15]. EGF increased the secretion of vascular endothelial growth factor, hepatocyte growth factor, and stromal-derived factor-1 via the ERK and JNK pathways in ASCs [16]. Epiregulin promotes the migration and chemotactic abilities of ASCs via the MAPK pathways [17]. However, the preconditioning effect of EGF mimetics, such as HB-EGF, on the hair regenerative potential of ASCs, has not been well-reported. Therefore, the present study investigated whether HB-EGF could enhance the hair regenerative potential of ASCs. The underlying signaling pathways and molecular mechanisms of ASC stimulation by these growth factors were clarified.

## 2. Results

### 2.1. HB-EGF Induces the Growth and Migration of ASCs

**Figure 1:**
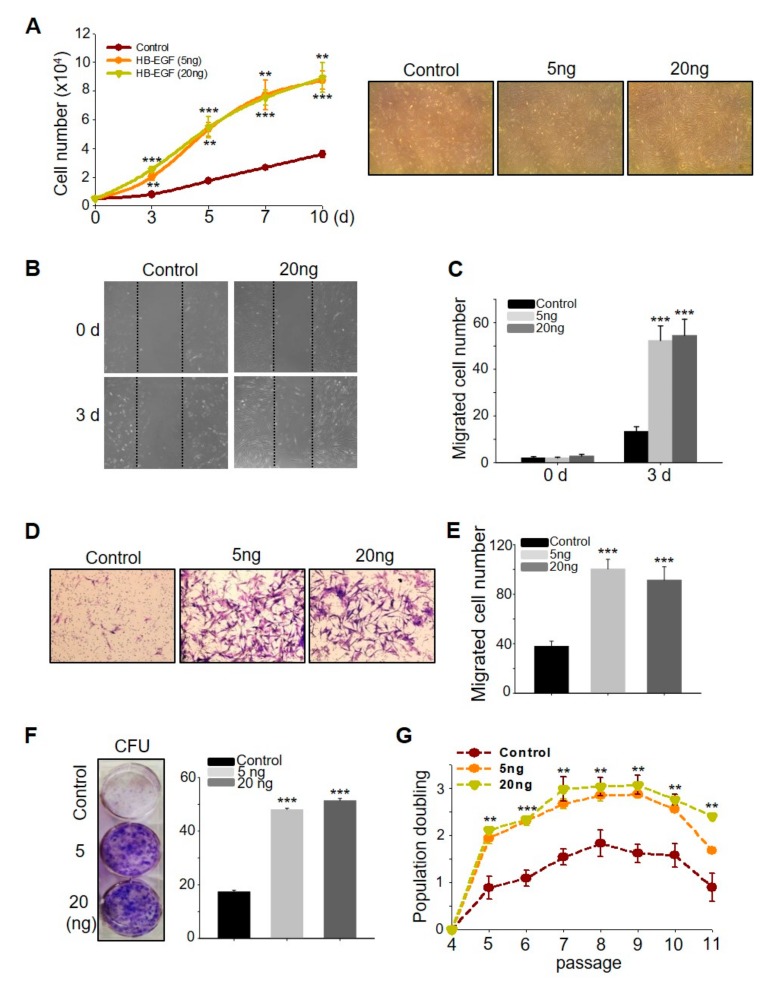
Heparin binding- epidermal growth factor (EGF)-like growth factor (HB-EGF) promotes adipose-derived stem cell (ASC) growth, migration, and self-renewal. (**A**) HB-EGF (5 and 20 ng) treatment induced ASC growth. HB-EGF (5 and 20 ng) treatment induced ASC migration, evidenced by the scratch migration assay (**B**,**C**) and transwell migration assay (**D**,**E**). (**F**) HB-EGF (5 and 20 ng) increased the colony-forming units, as evidenced by the clonogenic assay. (**G**) HB-EGF (5 and 20 ng) increased the population doubling rate. ** *p* < 0.01, *** *p* < 0.001. Three independent experiments were conducted for all data points. Error bars indicate the S.E.M.

To investigate whether HB-EGFs have stimulatory effects on ASCs, the number of live cells was monitored for 10 days post HB-EGF treatment. HB-EGF increased cell growth in a dose- and time-dependent manner (Figure 1A). HB-EGF also increased ASC migration dose-dependently, as evidenced by scratch and transwell migration assays (Figure 1B–E). To determine the self-renewal activity of HB-EGF, the colony-forming units (CFUs) were evaluated using a clonogenic assay and population doubling monitoring. HB-EGF increased both the CFUs and population doubling values of the ASCs (Figure 1F,G), indicating that the HB-EGF-treated cells exhibited self-renewal for a longer duration than the control cells. In addition, increased sudan III-positive cells in HB-EGF treated ASCs indicated that HB-EGF somewhat induced ASC differentiation into fat droplets (Supplementary Figure S1C). Cellular senescence, a potentially important contributor to cellular aging, was assessed with β-gal staining. In the late stage (passage 12), the number of β-gal-positive cells in the HB-EGF-treated groups was significantly lower than that in the control ASCs, indicating that HB-EGF-treated cells were less senescent than the control ASCs and were still able to proliferate at this late passage (Supplementary Figure S1A,B). Overall, these results indicated that HB-EGF increased the stimulatory effects and self-renewal activity of ASCs.

### 2.2. HB-EGF-Preconditioned ASCs Promote Hair Growth in Vivo

**Figure 2:**
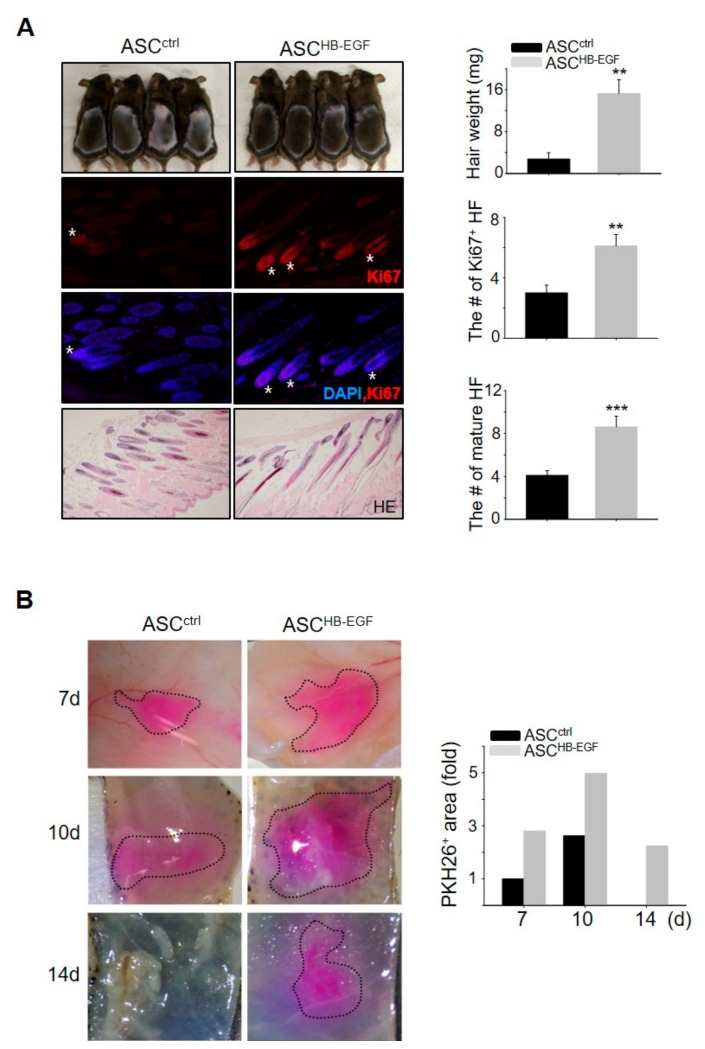
Preconditioning ASCs with HB-EGF promotes hair growth in vivo. (**A**) HB-EGF-preconditioned ASCs or untreated ASCs were injected into the dorsal skin of shaved mice. Images were captured, and the hair weight was measured 16–17 days later. Skin sections were analyzed using hematoxylin and eosin (HE) staining and the number of mature hair follicles (HF) was measured. Hair follicles with Ki67^+^ cells in the cortex were visualized by immunostaining. Asterisks indicate hair follicles with Ki67 ^+^ cells in cortex regions. ** *p* < 0.01, *** *p* < 0.001. Three independent experiments were conducted for all data points. *n* = 4 per group. Error bars indicate the S.E.M. (**B**) Preconditioned ASC^HB-EGF^ or ASC^ctrl^ were labeled with PKH26 red fluorescent dye; were injected into the dorsal skin of shaved mice; and were captured 7, 10, and 14 days later. Labeled cell area is marked with a black dotted line.

We examined whether ASCs preconditioned with HB-EGF can induce significant hair growth by performing a telogen-to-anagen induction assay. Preconditioning of the ASCs with HB-EGF induced robust hair growth, as evidenced by an increase in hair weight (Figure 2A). Hematoxylin and eosin (HE) staining and immunofluorescence staining for Ki67, which is a proliferating cell marker in the matrix amplifying zone of hair follicles, revealed that skin sections from ASC^HB-EGF^ mice exhibited a greater number of mature hair follicles and hair follicles with Ki67^+^ cells compared with the control group (Figure 2A). To identify human ASCs incorporated into mouse tissues, human-specific *ALU* and mouse *c-MOS* were amplified in purified genomic DNA from the back skin of mice. The identification of human *ALU* revealed that cultured human cells were incorporated into the back skin of the mice (Supplementary Figure S2). To trace the injected ASCs in vivo, preconditioned ASC^HB-EGF^ or ASC^ctrl^ were labeled with PKH26 red fluorescent dye and were injected into the dorsal skin of shaved mice, and images were taken at 7, 10, and 14 days (Figure 2B). The amount of PKH26-labeled cells in the HB-EGF treated groups was always high compared to the control group (Figure 2B). Even at day 14, PKH26-labeled cells in the control group had gone, while PKH26-labeled cells in the HB-EGF-treated groups still remained (Figure 2B). This result indicated that preconditioning of the ASCs with HB-EGF led to increased cell growth and survival during telogen-to-anagen induction. These results suggested that ASCs preconditioned with HB-EGF have a high cell survival potential, resulting in the promotion of hair growth.

### 2.3. Conditioned Medium of Preconditioned ASCs with HB-EGF Promotes Hair Growth In Vivo

**Figure 3:**
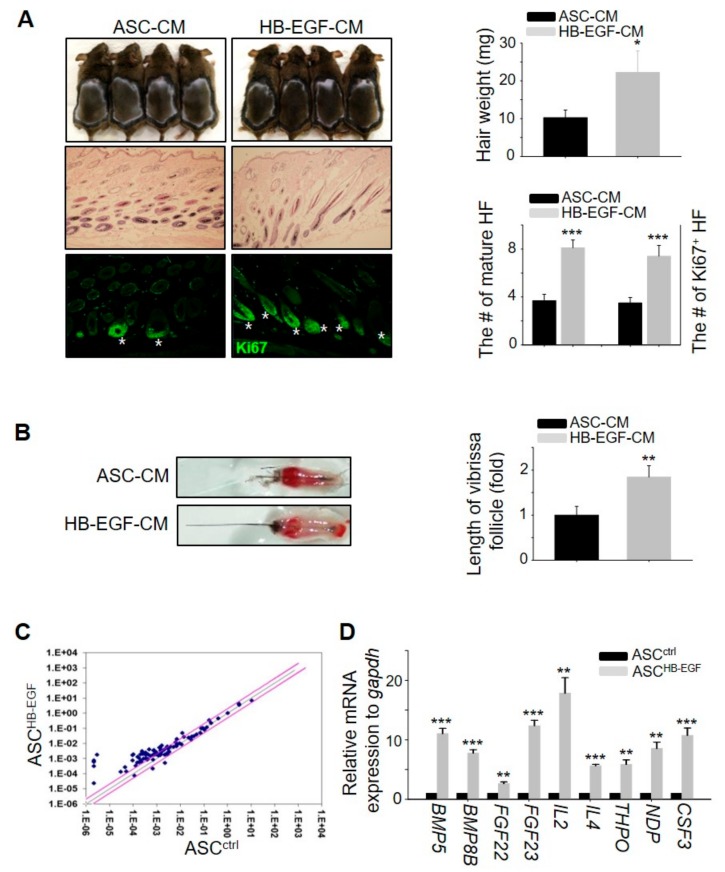
HB-EGF-conditioned medium (CM) promotes hair growth in vivo by releasing growth factors. (**A**) HB-EGF-CM was injected into the dorsal skin of shaved mice. Images were captured and hair weight was measured 16–17 days later. Skin sections were analyzed using HE staining and the number of mature hair follicles (HF) was measured. Hair follicles with Ki67^+^ cells in the cortex were visualized by immunostaining. Asterisks indicate hair follicles with Ki67^+^ cells in the matrix amplifying zone. *n* = 4 per group. Three independent experiments were conducted. (**B**) HB-EGF-CM increased the length of mouse vibrissal hair follicles compared with the control. (**C**) Upregulation of growth factors by HB-EGF was analyzed with a qPCR array to identify growth factors. Pink straight lines indicate ±2-fold change. (**D**) mRNA expression levels of nine genes were analyzed among the top notch genes from the qPCR array results. * *p* < 0.05, ** *p* < 0.01, *** *p* < 0.001. Three independent experiments were conducted for all data points. Error bars indicate the S.E.M.

As the HB-EGF-preconditioned ASCs induced hair growth (Figure 2), we investigated whether conditioned medium containing preconditioned ASCs with HE-EGF (HB-EGF-CM) also promoted hair growth in vivo. The results showed that HB-EGF-CM more rapidly induced telogen-to-anagen hair cycling, thereby promoting hair growth, evidenced by an increase in hair weight, the number of mature hair follicles, and the number of hair follicles containing Ki67^+^ cells (Figure 3A). Furthermore, treatment with HB-EGF-CM promoted the length of mouse vibrissal hair follicles in the organ culture (Figure 3B). To determine whether HB-EGF-CM released numerous growth factors to induce hair growth, a quantitative polymerase chain reaction (qPCR) array was performed for −90 growth factors. The expression levels of several growth factors in the HB-EGF-CM were markedly upregulated compared with those in the control group (Figure 3C), and the expression of nine genes among the upregulated growth factors was confirmed (Figure 3D). Overall, these results suggested that preconditioning ASCs with HE-EGF promoted hair growth by increasing the secretion of various growth factors.

### 2.4. Thrombopoietin Promotes Hair Growth by Stimulating DPCs

**Figure 4:**
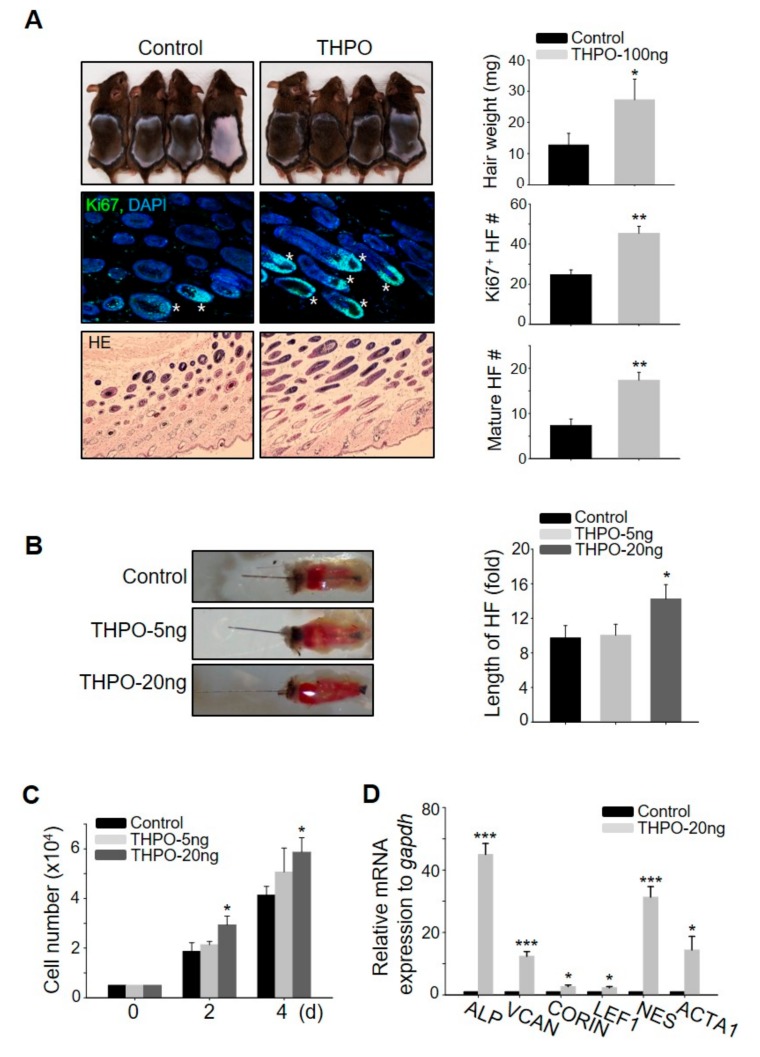
Thrombopoietin (THPO) injection promotes hair growth in vivo, stimulating dermal papilla cells (DPCs). (**A**) Recombinant human THPO was injected into the dorsal skin of shaved mice. The hair weights were measured, skin sections were analyzed by HE staining, and numbers of mature hair follicles and hair follicles with Ki67^+^ cells were measured 16–17 days later. Asterisks indicate hair follicles with Ki67^+^ cells in the matrix amplifying zone. *n* = 4 per group. Three independent experiments were conducted. (**B**) THPO increased the length of mouse vibrissal hair follicles compared with the control. (**C**) THPO increased DPC proliferation dose- and time-dependently. (**D**) THPO upregulated the mRNA expression of DPC marker genes in DPC. * *p* < 0.05, ** *p* < 0.01, *** *p* < 0.001. Three independent experiments were conducted per data point. All error bars indicate the S.E.M.

To elucidate which growth factors are key in triggering hair growth, the present study evaluated the telogen-to-anagen induction efficiency of five genes (BMP5, FGF23, IL2, THPO, and CSF3) among the nine top notch genes as the expression level of these five genes were >5-fold higher than in the control (Figure 3D). BMP5, FGF23, IL2, and CSF3 did not promote hair growth (Supplementary Figure S3), whereas the subcutaneous injection of THPO led to significant hair growth promotion, evidenced by telogen-to-anagen induction and the mouse vibrissal organ culture (Figure 4A,B). To investigate how the THPO released from ASCs triggers hair growth, the proliferation rate of DPCs was examined to assess whether THPO can stimulate DPCs. THPO treatment increased DPC growth dose- and time-dependently (Figure 4C) and the expression level of DPC marker genes, including alkaline phosphatase (ALP), versican (VCAN), CORIN, LEF1, nestin (NES), and actin alpha 1 (ACTA1) in vitro (Figure 4D). Overall, these results suggested that the preconditioning of ASCs with HB-EGF upregulates the expression of THPO and may lead to the secretion of THPO from ASCs for a longer period of time. Therefore, secreted THPO stimulates DPCs and transduces the signal for hair growth.

### 2.5. HB-EGF Increases ROS Levels by Regulating the Activity of NADPH Oxidase 4 (NOX4) in Mitochondria

**Figure 5:**
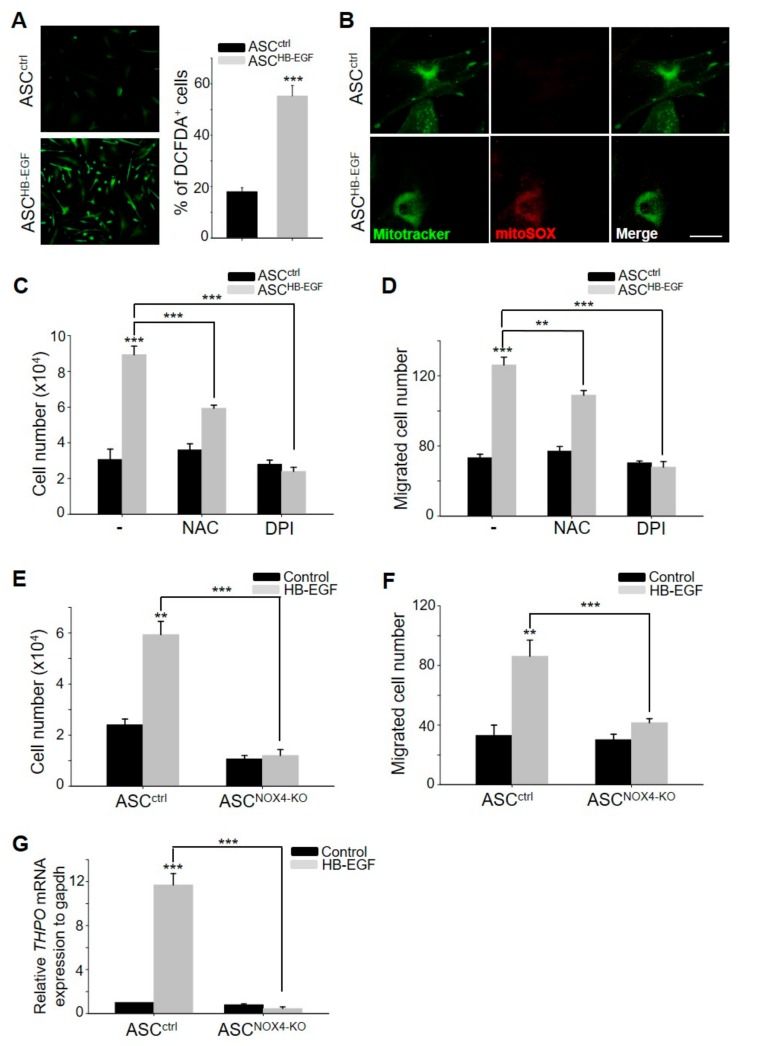
HB-EGF increases reactive oxygen species (ROS) levels by regulating NOX4 activity in mitochondria. (**A**) Cellular ROS levels were monitored following HB-EGF treatment using 2′,7′-Dichlorodihydrofluorescein diacetate (DCFDA) staining. (**B**) ROS levels in mitochondria were monitored using mitotracker and mito-SOX co-staining. Scale bar indicates 20 µm. (**C**,**D**) Increased cell growth and migration by HB-EGF were monitored following N-acetyl-L-cysteine (NAC) or diphenyleneiodonium (DPI) treatment. (**E**,**F**) Cell growth and migration following HB-EGF treatment were monitored in NOX4-knockout ASCs. (**G**) mRNA expression of THPO by HB-EGF was evaluated in NOX4-knockout ASCs by a quantitative polymerase chain reaction (qPCR) reaction. ** *p* < 0.01, *** *p* < 0.001. Three independent experiments were conducted for all data points. Error bars indicate the S.E.M.

Previous studies have revealed that NOX4 is primarily expressed in ASCs and plays a pivotal role in the hypoxia-enhanced stimulation of ASCs through the generation of ROS [18]. Therefore, to investigate whether the stimulation of ASCs by HB-EGF is associated with increased cellular ROS levels, the present study examined the cellular ROS levels and the production of ROS in mitochondria through 2′,7′-Dichlorodihydrofluorescein diacetate (DCFDA) staining and mitotracker/mito-SOX co-staining, respectively. HB-EGF treatment significantly increased the cellular ROS level (Figure 5A), and this increase was attributed to increased ROS production in mitochondria (Figure 5B). To examine whether increased cellular ROS levels can regulate ASC stimulation by HB-EGF, cell growth and migration were measured following N-acetyl-L-cysteine (NAC, an ROS scavenger) and diphenyleneiodonium (DPI, an NADPH oxidase inhibitor) treatment. NAC and DPI treatment attenuated the HB-EGF-induced increase in cell growth and migration (Figure 5C,D). As NOX4 is a key regulator of ROS levels among the NOX family (NOX1, NOX2, NOX3, and NOX5), the role of NOX4 in increased cell growth and migration by HB-EGF was investigated using NOX4-knockout ASCs. The cell growth and migration of the NOX4-knockout ASCs did not increase following HB-EGF treatment, whereas HB-EGF increased the growth and migration of the control ASCs (Figure 5E,F). In addition, the mRNA expression of THPO was not upregulated by HB-EGF in the NOX4-knockout ASCs, whereas the expression of THPO was significantly upregulated by HB-EGF in the control ASCs (Figure 5G). These results indicate that HB-EGF increased the production of ROS in mitochondria by regulating NOX4 activity. Moreover, NOX4 activity in ASCs can regulate THPO expression.

### 2.6. Hck Phosphorylation Pathway is Involved in the HB-EGF-Induced Stimulation of ASCs

**Figure 6:**
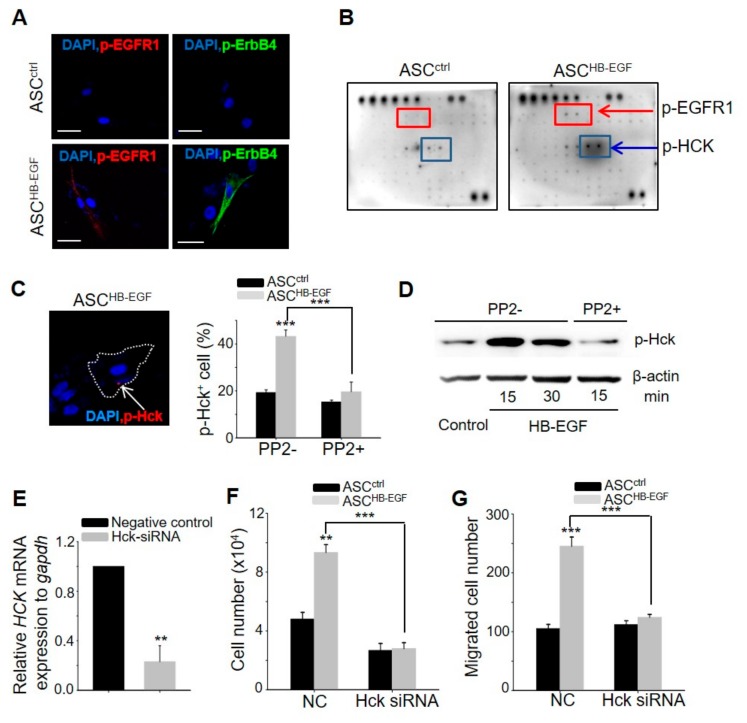
Hck phosphorylation pathway is involved in HB-EGF-induced stimulation of ASCs. (**A**) Expression of phospho-EGFR1 and phospho-ErbB4 following HB-EGF treatment. Phospho-EGFR1 and phospho-ErbB4 were present on the membranes of ASCs 10 min after HB-EGF (5 ng) treatment. No detection was shown in non-treated cells. Scale bars indicate 20 µm. (**B**) Expression of phospho-EGFR1 and phospho-Hck after HB-EGF treatment. The upregulation of phospho-EGFR1 and phospho-Hck 15 min after HB-EGF (5 ng) treatment was examined using a receptor tyrosine kinase array. (**C**) Phospho-Hck was present in the cytoplasm (arrow) in spot form and the percentage of phospho-Hck^+^ cells was evaluated. Scale bar indicates 20 µm. (**D**) Increased Hck phosphorylation by HB-EGF was examined by western blotting. (**E**) mRNA expression levels of Hck were downregulated in Hck-knockdown cells compared with negative control-treated cells. (**F**,**G**) Increased cell growth and migration by HB-EGF were monitored in Hck-knockdown ASCs. NC indicates negative control for siRNA. ** *p* < 0.01, *** *p* < 0.001. Three independent experiments were conducted per data point. Error bars indicate the S.E.M.

It is well-established that EGFR1 and ErBb4, which are receptor tyrosine kinases (RTKs), are receptors of HB-EGF, and their autophosphorylation elicits downstream activation and signaling [19]. HB-EGF induced the expression of phospho-EGFR1 and phospho-ErbB4 on the membrane of cultured ASCs at 10 min post-treatment (Figure 6A). To examine whether other RTKs besides EGFR1 and ErbB4 are associated with the HB-EGF-induced stimulation of ASCs, screening was performed via an RTK array. Notably, the expression of phospho-Hck was upregulated by HB-EGF treatment (Figure 6B), and this increase was confirmed using immunostaining and western blot analysis (Figure 6C,D). As Hck is an RTK and also a member of the Src kinase family, the present study examined whether PP2, an inhibitor of the Src kinase family, affects the phosphorylation of Hck by HB-EGF. The increased percentage of phospho-Hck-positive cells and the increase in the protein level of phospho-Hck induced by HB-EGF were inhibited by PP2 treatment (Figure 6C,D). The increased growth and migration induced by HB-EGF were also inhibited by PP2 treatment (Supplementary Figure S4). To elucidate more clearly the role of Hck in ASC stimulation by HB-EGF, the effect of Hck knockdown on ASCs (Figure 6E) was monitored. Increased cell growth and migration by HB-EGF were significantly inhibited by Hck knockdown (Figure 6F,G). These results indicated that HB-EGF stimulates ASCs through the phosphorylation of Hck.

## 3. Discussion

HB-EGF is a mitogenic and chemotactic molecule involved in tissue repair, tumor growth, and other tissue-modeling phenomena, including angiogenesis and fibrogenesis. Therefore, HB-EGF has been used to increase the proliferation and migration of mesenchymal stem cells (MSCs), which proliferate more rapidly and persistently in the presence of HB-EGF. This effect is dose-dependent and is inhibited by anti-HER-1 and anti-HB-EGF antibodies [20]. Ozaki et al. compared the chemotactic effects of growth factors and reported that EGF and HB-EGF showed mild stimulatory effects on MSCs [21]. HB-EGF has been shown to significantly increase the proliferation and migration of MSCs isolated from bone marrow and amniotic fluid [22]. HB-EGF also augments the ability of MSCs isolated from bone marrow and amniotic fluid to attenuate intestinal injury [23]. It is of importance that the present study is the first, to the best of our knowledge, to indicate that HB-EGF has mitogenic and preconditioning effects on ASCs.

The present study aimed to investigate the mechanism of action underlying the enhancing effects of HB-EGF on the hair growth-promoting effects of ASCs in vivo. Initially, PKH26-labeled hASCs were traced in vivo, and HB-EGF-preconditioned ASCs survived for longer periods of time than control ASCs after injection. In addition, HE-EGF increased hASCs growth by checking the PKH26^+^ cell area in vivo. These data were consistent with the increase in cell proliferation and population doubling in vitro. In addition, HB-EGF-CM exerted a superior hair growth-promoting effect on the ASC-CM. The present study analyzed the mRNA expression of growth factors and the results showed that diverse growth factors were upregulated following HB-EGF treatment. Therefore, it is reasonable to suggest that HB-EGF preconditioning enhanced the hair inductivity of ASCs via the migration to hair follicles following the secretion of some growth factors, specially THPO (thrombopoietin).

When we evaluated the hair growth effect of BMP5, FGF23, IL2, THPO, and CSF3, only THPO led to significant hair growth promotion in vivo and ex vivo. In addition, THPO stimulated DPCs that increase DPC growth and the upregulation of DPC marker genes. THPO is also known as a megakaryocyte growth and development factor, and regulates the differentiation of megakaryocytes and platelets. However, there is currently no study on THPO and hair growth. It was only reported that there could be an association between pulmonary hypertension and the THPO level [24]. THPO may be an important place for megakaryocytopoiesis in blood vessels [24]. Interestingly, vasodilators such as minoxidil and udenfil stimulated ASC motility, thereby inducing hair growth [25,26]. The relationship between the THPO level, vasodilation, and hair growth should be further studied. In addition, the mRNA expression of THPO was not upregulated by HB-EGF in the NOX4-knockout ASCs, whereas the expression of THPO was significantly upregulated by HB-EGF in the control ASCs (Figure 5G). These results indicate that a hypoxic condition can regulate THPO expression via NOX4 activity in ASCs. It has been reported that growth factors, including VEGF, PDGF, and EGF, are activated or secreted in a hypoxic state [27,28]. Although we did not check that hypoxia can induce THPO expression, an increased ROS level, which is a mimic condition with hypoxia in ASC by HB-EGF treatment, may induce the expression and release of THPO.

It has been reported that growth factors mediate increased proliferation, migration, and paracrine effects on ASCs via ROS generation [8]. For example, PDGF-B increased the proliferation and migration of ASCs via ROS generation. PDGF-D also induced the mitogenic effects of ASCs via ROS generation, and the preconditioning of ASCs with PDGF-D increased the paracrine effects of ASCs, enhancing the hair inductivity of ASCs [8]. EGF and its mimetics reportedly affect the proliferation and differentiation of ASCs [15] and EGFRs mediate these functions via ROS generation in other cell types [29]. However, this is the first indication that HB-EGF increases the mitogenic and hair growth-promoting effects of ASCs via ROS generation. The ROS-generating system was further examined and the results showed that HB-EGF led to ROS generation in the mitochondrial region in ASCs. The expression of NOX4 is high in the mitochondria of ASCs, and NOX4 silencing attenuated the stimulation by HB-EGF, indicating that HB-EGF increases ROS generation through mitochondrial NOX4 in ASCs.

It has been well-established that EGF mimetics increase cell proliferation and migration through Src family activation. For example, the EGF-dependent activation of Src family tyrosine kinases has been observed in NIH3T3 and A431 cells [30]. The inhibition of Src family kinases by PP1 inhibits the EGF-induced activation of Akt, phosphorylation of c-Cbl, and ubiquitination of EGFR [31]. However, there is little to no direct evidence that HB-EGF mediates mitogenic signals through the phosphorylation of Hck in ASCs. In the present study, it was found that HB-EGF phosphorylates Hck, which subsequently phosphorylates Akt and Erk. The siRNA-mediated knockdown of Hck attenuated the HB-EGF-mediated proliferation and migration of ASCs.

**Figure 7:**
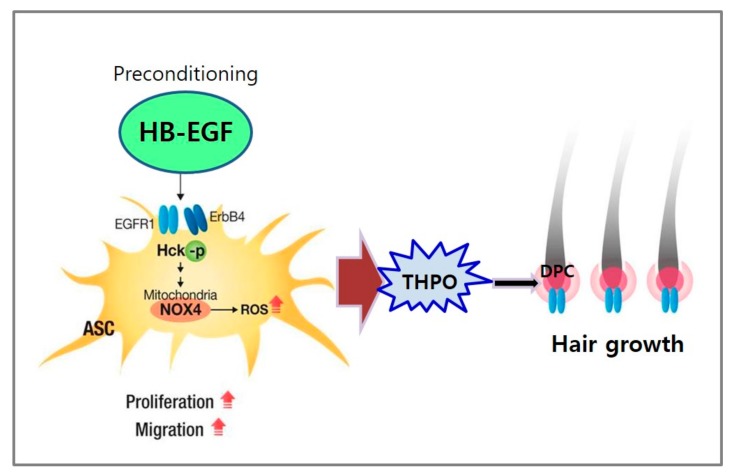
Summary. Preconditioning of ASCs with HB-EGF stimulates ASCs via ROS generation by NOX4 and Hck phosphorylation. Therefore, THPO released from ASCs stimulates hair growth by stimulating DPCs.

In summary, HB-EGF increased the proliferation, migration, and growth factor secretion of ASCs via ROS generation and the phosphorylation of Hck (Figure 7). The preconditioning of ASCs with HB-EGF improved the hair growth-promoting effects of ASCs via the secretion of growth factors such as THPO. Thus, released THPO from ASCs stimulated DPCs, thereby transducing signals for hair growth. Therefore, combination therapy consisting of HB-EGF and ASCs may offer a novel solution for hair-loss treatment.

## 4. Materials and Methods

### 4.1. Cell Culture

Human ASCs were isolated via the liposuction of subcutaneous fat, as reported previously [32,33], following informed consent (Yonsei University College of Medicine, 4-2018-0141). The ASCs were cultured in α-MEM (α-minimum essential medium, Hyclone, Logan, UT, USA) with 10% FBS (fetal bovine serum, Gibco, CA, USA) and 1% penicillin/streptomycin (Gibco). Human DPCs were purchased from PromoCell (#C-12071) and cultured in follicle DPC medium with supplement mix (PromoCell, Heidelberg, Germany) and 0.1% anti-antibiotics (Gibco). DPCs and ASCs were maintained at 37 °C in a humidified 5% CO_2_ incubator. DPCs and ASCs for all experiments were used at passages 2–3 and approximately passages 4–6, respectively.

### 4.2. Cell Growth Assay

To measure cell growth, the cells were seeded in 12-well plates at 5 × 10^3^ cells per well, treated with HB-EGF (ASCs, 5 or 20 ng) and THPO (DPC, 5 or 20 ng), and incubated for 10 and 4 days, respectively. To measure the cell number, cells were then trypsinized (Gibco, CA, USA), stained with trypan blue (Sigma-Aldrich, MO, USA), and counted each day using a hemocytometer under a Nikon ECLIPSE Ts2 microscope.

### 4.3. Scratch Migration Assay

To measure the migratory effect, the cells were seeded into 6-well plates, treated with HB-EGF (5 and 20 ng) after 24 h, and cultured to confluence. A sterile 1-mL pipette tip was used to scratch the cell monolayer. The cells were then washed with PBS, treated again with HB-EGF (5 and 20 ng) in serum-free medium, and incubated for 4 days. The migration of cells into the scratched area (wound closure) was visualized using a ZEISS Observer D1 microscope. Multiple images were captured per well and monitored over 4 days. The average cell number was counted using the Adobe photoshop CS6 extended program.

### 4.4. Cell Migration Measurement Using a Transwell Migration Assay

The ASCs were seeded into 60-mm plates (5 × 10^4^ per well) and treated with HB-EGF (5 and 20 ng) for 3 days. The cells (1.5 × 10^4^ per well) were then cultured in serum-free medium for 24 h and seeded on the upper side of transwell membrane plates (BD Falcon, CA, USA). Next, 700 μL medium containing serum was added to the lower chambers. The cultures were incubated for 24 h to allow transwell migration. To remove non-migrated cells, the upper surface of inserts was cleaned with cotton swabs and washed with PBS. The inserts were stained with 0.1% formalin/2% crystal-violet solution (Sigma-Aldrich) for 30 min and dried. Multiple random images (10) per insert were captured using a ZEISS Observer D1 microscope and the average number of cells was counted using the Adobe photoshop CS6 extended program.

### 4.5. Measurement of Colony-Forming Units (CFUs)

For the clonogenic assay, 1 × 10^3^ ASCs were seeded in six-well plates, treated with HB-EGF (5 or 20 ng) after 24 h, and incubated for 14 days. The cells were stained with formalin/0.1% crystal violet solution and analyzed using a Nikon ECLIPSE Ts2 microscope. 

### 4.6. Measuring Population Doublings

To measure the population doublings of the ASCs following HB-EGF treatment, cells at passage 4 were seeded in 6-well plates at 5 × 10^4^ cells per well, followed by the addition of HB-EGF the next day and incubation for 5 days. The cells were trypsinized, stained with trypan blue, and counted using a hemocytometer. This step was repeated up to seven times (until passage 11). The numbers of cells seeded at the start of each passage and harvested at the end were used to calculate the number of population doublings. The formula for population doublings (PD) is as follows: PD = ln (harvested cells/seeded cells)/ln 2, where PD = population doublings and ln = natural log.

### 4.7. RNA Extraction, Quantitative RT-PCR, qPCR Array, and RT-PCR

Total RNA was extracted from the ASCs using TRIzol reagent (Invitrogen, NY, USA). cDNA was synthesized using extracted RNA, oligodT, and the HelixCript™ Thermo Reverse Transcription system (NANOHELIX, WI, USA), according to the manufacturer’s instructions. SYBRGreen qPCR master mix (Takara, Shiga, Japan) was used for qPCR reactions, according to the manufacturer’s instructions. For the qPCR reaction, total RNA was extracted from HB-EGF (5 ng)-treated ASCs, THPO (20 ng)-treated DPCs, and NOX4-KO ASCs, and subjected to cDNA synthesis in the manner described above. The qPCR array for growth factors was conducted using an RT² First Strand cDNA Synthesis Kit (Cat#: PAHS-041ZC, QUAZEN, Hilden, Germany). For identification of the human *ALU* and mouse *c-MOS* genes, genomic DNA was isolated from human ASCs and mouse back skin tissues using a genomic DNA isolation kit (Bioneer, Daejeon, Korea). The RT-PCR conditions were as follows: 95 °C for 5 min, followed by 40 cycles of 95 °C for 1 min, 55 °C for 1 min, 72 °C for 1 min, and 72 °C for 10 min. The primers of human *ALU* and mouse *c-MOS* are described in Supplementary Table S1.

### 4.8. X-Gal Staining for Cellular Senescence and Sudan III Staining

A β-galactosidase assay was carried out using a β-galactosidase staining kit (Sigma-Aldrich) to assess cellular senescence, according to the manufacturer’s instructions. In summary, aged cells were fixed with fixation buffer (20% formaldehyde and 2% glutaraldehyde in PBS) for 10 min and washed with PBS. The fixed cells were incubated at 37 °C overnight, following the addition of cell-staining solution containing potassium ferricyanide, potassium ferrocyanide, and X-gal. The following day, the cell-staining solution was removed and the cells were washed twice with PBS. Green-colored cells were considered senescent. For staining, fat droplet, control, or HB-EGF-treated ASCs were stained with sudan III (Sigma-Aldrich, MO, USA).

### 4.9. Hematoxylin and Eosin Staining

For hematoxylin and eosin staining, paraffin sections were dewaxed using xylene for 15 min three times and hydrated in 100%, 90%, 80%, and 70% EtOH. Then, slides were dipped into Mayer’s hematoxylin (Sigma-Aldrich) for 10 min and rinsed in flowing water for 1 min. Slides was again dipped into eosin Y (Sigma-Aldrich) for 1 min 30 sec and rinsed in flowing water for 1 min. Slides were dehydrated with 70%, 80%, 90%, and 100% EtOH; washed with fresh xylene for 15 min two times; and then dried and mounted with mounting medium. Images were captured using a ZEISS Observer D1 microscope.

### 4.10. Immunofluorescence Staining

The paraffin sections were dewaxed using xylene for 15 min three times and hydrated in 100%, 90%, 80%, and 70% EtOH. Antigen retrieval was performed by a microwave in boiling antigen retrieval solution (pH 6.0, Dako, CA, USA) for 2 min 20 s. The sections were stained with the rabbit Ki67 antibody (Abcam, IA, USA, 1:300) overnight at 4 °C, and then incubated with secondary antibodies, such as Alexa Fluor 594 goat anti-rabbit IgG or Alexa Fluor 488 goat anti-rabbit IgG (Invitrogen, NY, USA, 1:1000), for 1 h at room temperature with 4,6-diamidino-2-phenylindole (DAPI; Sigma-Aldrich). For cell staining with phospho-EGFR1, phospho-ErBb4, or phospho-Hck antibodies, the cells were fixed with 4% paraformaldehyde for 30 min at room temperature; washed with PBS; and incubated with phospho-EGFR1 (Abcam, 1:300), phospho-ErBb4 (Abcam, 1:300), or phospho-Hck (Abcam, 1:300) antibodies overnight at 4 °C. The samples were then incubated with Alexa Fluor 594 goat anti-rabbit IgG or Alexa Fluor 488 goat anti-rabbit IgG (Invitrogen, NY, USA, 1:1000) secondary antibodies for 1 h at room temperature with DAPI. Images of immunofluorescence staining were captured using a ZEISS LSM700 confocal microscope.

### 4.11. Western Blot Analysis

The cells were lysed with protein extraction solution (PRO-PREP™; iNtRON, Seoul, Korea) containing protease inhibitors and western blot analysis was performed as follows. The protein extracts were loaded on an 8% acrylamide gel, blotted on a nitrocellulose membrane, and incubated with the phospho-Hck antibody (Abcam, IA, USA, 1:500) overnight at 4 °C. The membrane was then washed with TBST buffer and incubated with HRP-tagged secondary antibodies (Jackson ImmunoResearch, PA, USA) for 1 h. Blot images were obtained using ImageQuant LAS 4000 (GE Healthcare Life Sciences, PA, USA).

### 4.12. Mouse Anagen Induction

The mice were maintained and anesthetized according to a protocol approved by the U.S. Pharmacopoeia and the Institutional Animal Care and Use Committee of Yonsei University (IACUC-201712-681-03, 22 October 2018: IACUC-201902-866-01, 11 March 2019). The dorsal region of 6-week-old male C3H/HeN mice in the telogen stage of the hair cycle was shaved with a clipper and electric shaver, with special care taken to avoid damaging the bare skin. Subsequently, 5 × 10^4^ of the control ASCs or ASCs treated with HB-EGF for 48 h were injected into the dorsal skin of the shaved mice, as indicated. Conditioned medium from the ASCs or HB-EGF-treated ASCs was injected into the dorsal skin of the shaved mice. Any darkening of the skin (indicative of hair cycle induction) was carefully monitored by image capture. After approximately 16–17 days, the dorsal hair was shaved and weighed to estimate the growth rate, and the dorsal skin was analyzed using HE staining and immunostaining. Human THPO peptide (100 ng/per day/1 male, PeproTech, CA, USA) was injected into the dorsal skin of the shaved mice and also analyzed with the same method above mentioned. 

### 4.13. Purification of Conditioned Medium and Vibrissae Follicle Organ Culture

For purification of the conditioned medium of control and HB-EGF-treated ASCs, the ASCs were seeded in a 100-mm plate at a density of 1 × 10^6^. After 24 h, HB-EGF (5 ng) was added and cultured with normal medium for 2 days, and then cultured with serum-free medium for 1 day. The medium was filtered with a 0.2-µm membrane and was centrifuged in a VIVASPIN at 4 °C. For anagen induction, 100 µL conditioned medium was injected into the dorsal skin of the shaved mice. For organ culture, vibrissae hair follicles were cut from the vibrissae hair of C57BL/6 mice and washed with PBS on ice. Then, vibrissae hair follicles were cultured in special medium (Williams E medium supplemented with 2 mM l-glutamine, 10 µg mL^−1^ insulin, 10 ng mL^−1^ hydrocortisone, 1% penicillin/streptomycin, without serum) with the conditioned medium, THPO peptide (5 or 20 ng), for 3 days.

### 4.14. Statistical Analysis

Three independent experiments were conducted for all data points. All data are presented as the mean ± standard error. The mean values were compared using the Student’s t-test. For all statistical tests, *p* < 0.05 was accepted as statistically significant.

## Supplementary Materials

Supplementary materials can be found at https://www.mdpi.com/1422-0067/21/1/122/s1.

## Abbreviations

ASC	Adipose-derived stem cell
THPO	Thrombopoietin
PDGF-D	Platelet-derived growth factor-D
DPC	Dermal papilla cell
bFGF	basic fibroblast growth factor
NAC	following N-acetyl-L-cysteine
DPI	diphenyleneiodonium
ROS	reactive oxygen species
