# Supplementary material for: HB-EGF Improves the Hair Regenerative Potential of Adipose-Derived Stem Cells via ROS Generation and Hck Phosphorylation
Source: Int J Mol Sci. 2019 Dec 23;21(1):122. doi: 10.3390/ijms21010122 (PMC6981845; doi:10.3390/ijms21010122)
Supplement: Supplementary file 1 [file ijms-21-00122-s001.pdf]

Supplementary Materials:

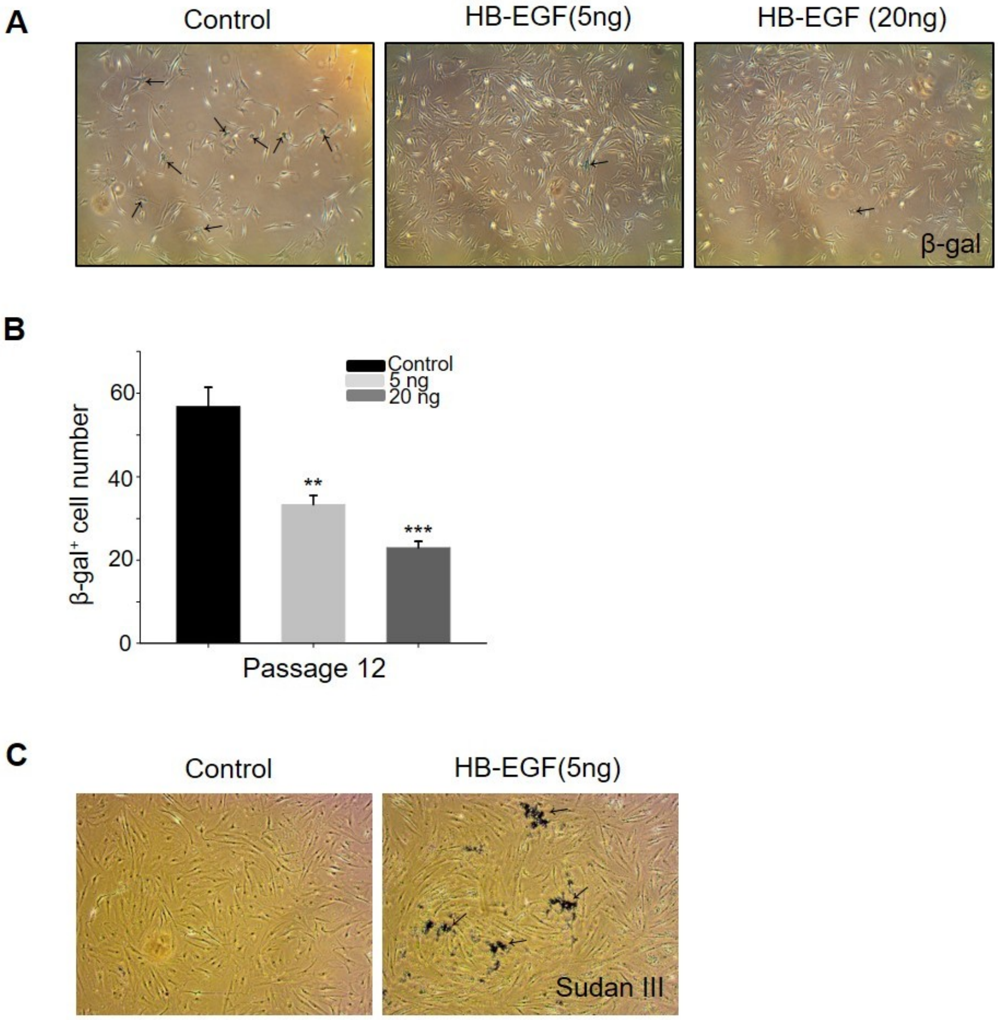

Supplementary figure 1. Preconditioned ASCs with HB-EGF are resistant against cellular senescence. **(A)** Preconditioning of ASCs with HB-EGF decreased the number of β-gal$^+$ cells (arrow), even at passage 12. **(B)** Graph showing the number of β-gal$^+$ cells. Three independent experiments were conducted per all data point. All error bars indicate the S.E.M. **(C)** Sudan III staining is visible in HB-EGF treated cells.

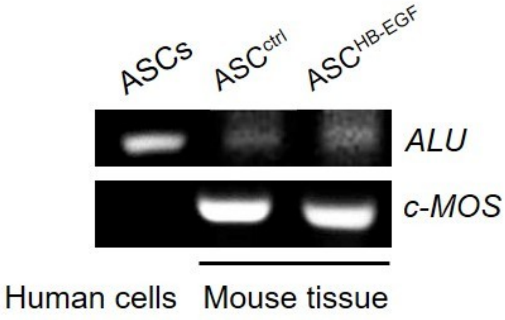

Supplementary figure 2. Identification of human *ALU* in injected skin tissues of mice.
The human-specific *ALU* and mouse *c-MOS* were amplified in purified genomic DNA from back skin of ASC^{ctrl}, ASC^{epiregulin}, ASC^{HB-EGF} mice and cultured human ASCs.

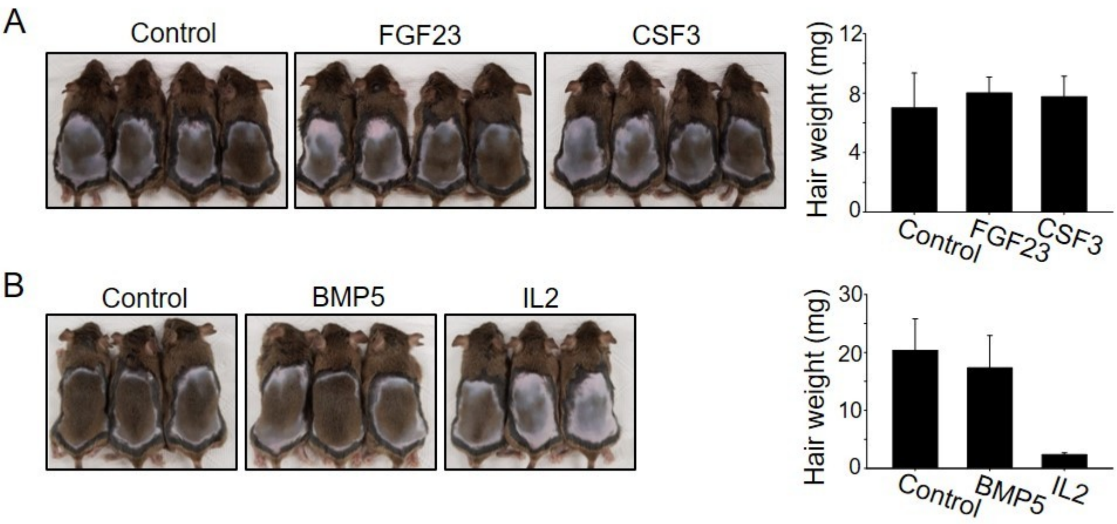

Supplementary figure 3. Effect of preconditioned ASCs with growth factors on hair growth *in vivo*.
(A) FGF23- or CSF3-preconditioned ASCs, **(B)** BMP5- or IL2-preconditioned ASCs or untreated ASCs were injected into the dorsal skin of shaved mice. Images were captured, and hair weights measured 16-17 days later. n=3-4 per group. All error bars indicate the S.E.M.

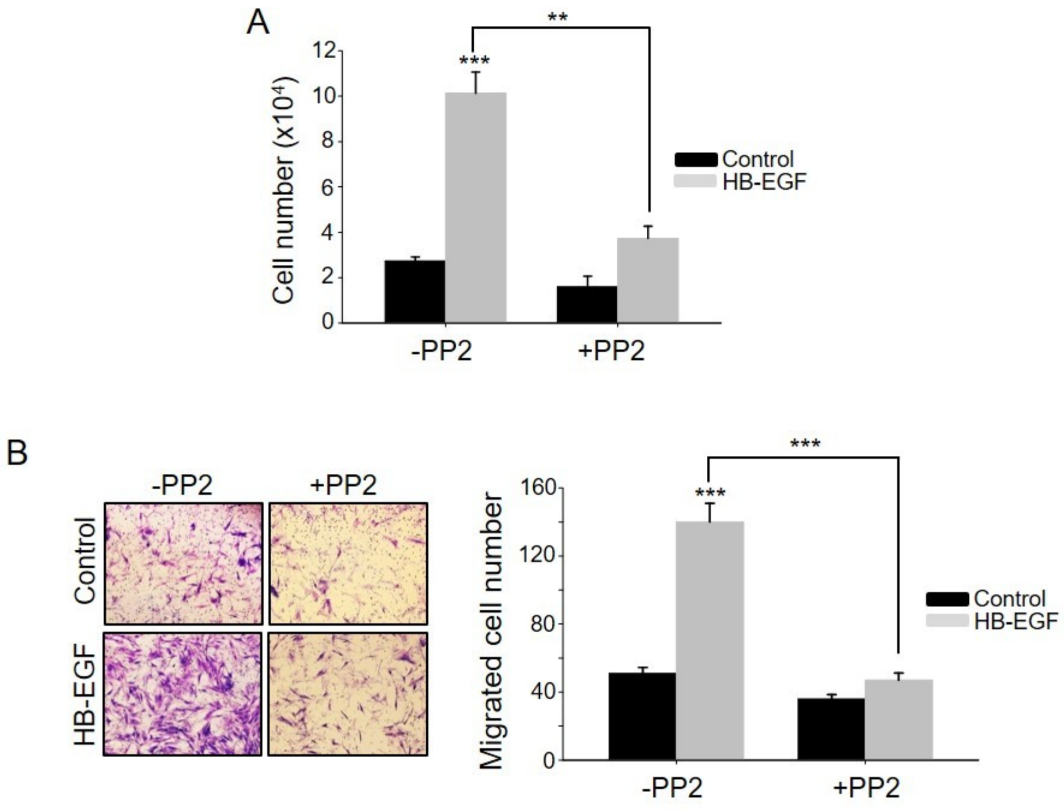

Supplementary figure 4. Effect of src kinase inhibitor on ASC growth and migration by epiregulin or HB-EGF. **(A)** ASC growth was monitored after HB-EGF (5 ng) treatment until 6 days in PP2 (src kinase inhibitor)-treated or -untreated group. ***$p < 0.001$. Three independent experiments were conducted per all data point. All error bars indicate the S.E.M. **(B)** ASC migration was examined after HB-EGF (5 ng) treatment in PP2-treated or -untreated group. ***$p < 0.001$. Three independent experiments were conducted per all data point. All error bars indicate the S.E.M.

Supplementary Table 1; Primers used for QPCR and PCR

Gene	Primer sequences (5′-3′)
AREG	GRGGRGCTGTCGCTCTTGATA<br>CCCCAGAAAATGGTTCACGCT
BPM5	GCTGCTGGGTTCTAGTGGG<br>TTCGTGGTTCCGTAGTCTTCTA
BMP8B	GGGAGCGCATCGAAGAGAAC<br>CTGTGAGGCGTAGGTGTTGTG
CSF3	GCTGCTTGAGCCAACTCCATA<br>GAACGCGGTACGACACCTC
EGFR1	AGGCACGAGTAACAAGCTCAC<br>ATGAGGACATAACCAGCCACC
ErbB4	GTCCAGCCCAGCGATTCTC<br>AGAGCCACTAACACGTAGCCT
FGF22	GGGAGCGCATCGAAGAGAAC<br>CTGTGAGGCGTAGGTGTTGTG
FGF23	CAGAGCCTATCCCAATGCCTC<br>GGCACTGTAGATGGTCTGATGG
GAPDH	CGAGATCCCTCCAAAATCAA<br>TGTGGTCATGAGTCCTTCCA
HB-EGF	ATCGTGGGGCTTCTCATGTTT<br>TTAGTCATGCCCAACTTCACTTT
HCK	CCCTGTATGATTACGAGGCCA<br>CACTCCCCGGATTCCTCTAGG
Human-ALU	CAGGACCTGAGAAAGGACACTATCC<br>CAAACAAGAGGCACACTTTCAACCA
IL2	AACTCCTGTCTTGCATTGCAC<br>GCTCCAGTTGTAGCTGTGTTT
IL4	CGGCAACTTTGTCCACGGA<br>TCTGTTACGGTCAACTCGGTG
Mouse-c-mos	GAATTCAGATTTGTGCATACACAGTGACT<br>AACATTTTTCGGGAATAAAAGTTGAGT
NDP	ACAGTAAAACGGACAGCTCATTC<br>GGTTGCTTGAGGACAGTGC
THPO	AACTGCAAGGCTAACGCTGT<br>GACATGGGAGTCACGAAGCA
